# Precise Fecal Microbiome of the Herbivorous Tibetan Antelope Inhabiting High-Altitude Alpine Plateau

**DOI:** 10.3389/fmicb.2018.02321

**Published:** 2018-09-28

**Authors:** Xiangning Bai, Shan Lu, Jing Yang, Dong Jin, Ji Pu, Sara Díaz Moyá, Yanwen Xiong, Ramon Rossello-Mora, Jianguo Xu

**Affiliations:** ^1^State Key Laboratory for Infectious Disease Prevention and Control, Collaborative Innovation Center for Diagnosis and Treatment of Infectious Diseases, National Institute for Communicable Disease Control and Prevention, Chinese Center for Disease Control and Prevention, Beijing, China; ^2^Marine Microbiology Group, Department of Ecology and Marine Resources, Mediterranean Institute for Advanced Studies, Spanish National Research Council (CSIC)-University of the Balearic Islands (UIB), Esporles, Spain; ^3^Shanghai Public Health Clinical Center, Fudan University, Shanghai, China

**Keywords:** microbiome, full-length 16S rRNA gene, Tibetan antelope, metataxonomics, operational phylogenetic unit, high-altitude, herbivorous

## Abstract

The metataxonomic approach combining 16S rRNA gene amplicon sequencing using the PacBio Technology with the application of the operational phylogenetic unit (OPU) approach, has been used to analyze the fecal microbial composition of the high-altitude and herbivorous Tibetan antelopes. The fecal samples of the antelope were collected in Hoh Xil National Nature Reserve, at an altitude over 4500 m, the largest depopulated zone in Qinghai-Tibetan Plateau, China, where non-native animals or humans may experience life-threatening acute mountain sickness. In total, 104 antelope fecal samples were enrolled in this study, and were clustered into 61,258 operational taxonomic units (OTUs) at an identity of 98.7% and affiliated with 757 OPUs, including 144 known species, 256 potentially new species, 103 potentially higher taxa within known lineages. In addition, 254 comprised sequences not affiliating with any known family, and the closest relatives were unclassified lineages of existing orders or classes. A total of 42 out of 757 OPUs conformed to the core fecal microbiome, of which four major lineages, namely, un-cultured *Ruminococcaceae, Lachnospiraceae, Akkermansia*, and *Christensenellaceae* were associated with human health or longevity. The current study reveals that the fecal core microbiome of antelope is mainly composited of uncultured bacteria. The most abundant core taxa, namely, uncultured *Ruminococcaceae*, uncultured *Akkermansia*, uncultured *Bacteroides*, uncultured *Christensenellaceae*, uncultured *Mollicutes*, and uncultured *Lachnospiraceae*, may represent new bacterial candidates at high taxa levels, and several may have beneficial roles in health promotion or anti-intestinal dysbiosis. These organisms should be further isolated and evaluated for potential effect on human health and longevity.

## Introduction

The Tibetan antelope (*Pantholops hodgsonii*) is endemic to the Qinghai Tibetan Plateau in China. The plateau is characterized by open, highly elevated alpines and desert steppe habitats on a mountainous terrain at elevations between 3,250 and 5,500 m, with a low partial pressure of oxygen and a high level of ultraviolet radiation (Zhou et al., 2007; Zhuge et al., 2015). Non-native animals or humans visiting such regions may experience life-threatening acute mountain sickness, which is a pathological effect of high altitude on humans, caused by acute exposure to a low partial pressure of oxygen. This condition resembles cases of flu, carbon monoxide poisoning, or a hangover, which can progress to fatal high altitude pulmonary edema or high altitude cerebral edema. However, the Tibetan antelopes, which have survived for millions of generations on the plateau, are well adapted to this environment and can run at up to 80 km per hour under this low oxygen condition (Ge et al., 2013). Analysis of the draft genome has identified common themes of positive selection involved in the evolution of genes that respond to hypoxia, energy metabolism, and oxygen transmission (Ge et al., 2013). Tibetan antelopes feed on forbs, grasses, and sedges.

In the current study, we have analyzed the fecal microbiomes of Tibetan antelopes residing in several different locations of the Hoh Xil National Nature Reserve of the Qinghai Province, where besides the high altitude, the climate is extremely fierce and inferior, which is not suitable for human activities. Here, we used the metataxonomics methodology already published using PacBio Technology (Meng et al., 2017) to study the fecal microbiome of Tibetan antelopes. The use of this technology for amplicon diversity studies combined with applying the OPU (Mora-Ruiz Mdel et al., 2015; Vidal et al., 2015), has two advantages of (i) using almost full-length 16S rRNA gene sequences to yield higher taxonomic resolution at the genus and species level, and (ii) allowing a *de novo* tree reconstruction to increase reliability and accuracy. The results in this study, to our knowledge, are the first in revealing high-mountain ruminant fecal microbiomes using a high-resolution metataxonomic approach.

## Materials and Methods

### The Sampling of Antelope Feces and Ethical Statement

The antelope fecal samples were collected from Hoh Xil National Nature Reserve, Qinghai Province, China, in August of 2014. Tibetan antelopes were spotted while driving along the Qinghai-Tibetan Highway, the freshly excreted feces were localized by using telescope. Feces were immediately collected after antelopes moved away from their stool. A total of 104 fecal samples were collected from four sites, designated A to D (**Table 1**). Thirty-two samples were obtained from site A at an altitude of 4514.4 m (35°37′ N, 93°44′ E), 16 were obtained from site B at an altitude of 4561.7 m (35°51′ N, 93°73′ E), 29 samples were obtained from site C at an altitude of 4614.6 m (35°55′ N, 93°86′ E); and 27 samples were from site D at an altitude of 4693.5 m (35°59′ N, 94°03′E). The feces were collected into sterile tubes, placed in icebox immediately and brought to the microbiological laboratory of local Center for Diseases Control and Prevention of Qinghai Province. Samples were finally stored at -20°C with Uninterruptible Power System, to transport to our laboratory in Beijing.

**Table 1:** Number of reads, OTUs, and OPUs from the four sampling sites.

Site	Number of samples	Number of reads	Number of OTUs	Number of OPUs	Location (above m. s. l, latitude/longitude)
A	32	309,227	60,996	559	4514.4 m, 35°37′ N/93°44′ E)
B	16	104,174	60,442	329	4561.7 m, 35°51′ N/93°73′ E)
C	29	209,813	60,594	373	4614.6 m, 35°55′ N/93°86′ E)
D	27	244,220	60,880	502	4693.5 m, 35°59′ N/94°03′ E)
Total	104	867,434	–^a^	–^b^

*^*a*^61,258 unique OTUs were clustered for 104 samples. ^*b*^757 unique OPUs were assigned in total.*

Antelope fecal samples were picked up from the ground after herds of antelope passing through, which was non-invasive. Wildlife Protection Agents of Qinghai Province approved the animal welfare practice associated with this study. In addition, the study was approved by ethics committee of National Institute for Communicable Disease Control and Prevention, China CDC, according to Chinese ethic laws and regulations, with number ICDC-2016004.

### Almost Full Length 16S rRNA Gene Amplicon Sequencing Using PacBio System

Genomic DNA was extracted from feces using QIAamp Fast DNA Stool Kit (Qiagen, Hilden, Germany). The full V1-V9 region of the bacterial 16S rRNA gene was amplified using the universal primer set 27F (5′-AGAGTTTGATCCTGGCTCAG-3′) and 1492R (5′-GNTACCTTGTTACGACTT-3′) (Weisburg et al., 1991). Sequencing was conducted on PacBio RS II platform at TianJin Biochip Corporation, China. Primary 16S rRNA sequences generated were filtered using the SMRT Portal (version 2.3.0). ^1^ To ensure assignment of the barcoded reads to their original samples correctly, a minimum barcode score of 22 was selected to achieve an accuracy of 99.5%. Data containing ambiguous bases were removed, primer sequences and adapters were excised from the filtered reads, and sequences outside of nucleotide positions 10–1490 were trimmed. Qiime (Bokulich et al., 2013) was applied for further filtering. Chimeras were removed using the UCHIME algorithm and RDP Gold was used as reference database (Wang et al., 2007; Edgar et al., 2011). Rarefaction curves using PAST software (v3.15)^2^ (Hammer Y. et al., 2001) were calculated as previously described (Mora-Ruiz Mdel et al., 2016). The sequencing error rate was determined for each sample as previously described (Schloss et al., 2016). The insertion and deletion rates were obtained by comparing the final alignment of all representative selected sequences with the corresponding reference sequence in the LTP123 and SILVA REF NR databases. The microbiome raw sequence data of this study have been deposited in the NCBI Sequence Read Archive (SRA) with the accession number SRR5768980.

### The Operational Phylogenetic Unit (OPU) Analyses Strategy

All the full-length 16S rRNA sequences were clustered into OTU with an identity threshold set at 98.7% using the USEARCH pipeline (Edgar, 2013). The most frequent representative sequences of each OTU were selected for a phylogenetic inference using the LTP123 database (Yarza et al., 2010) or the SILVA REF NR database (Quast et al., 2013). The OPUs were designed based on the visual inspection of the final tree as previously described (Mora-Ruiz Mdel et al., 2015; Vidal et al., 2015). One OPU was defined as the smallest monophyletic clade formed by query sequences and a reference sequence from the databases, including the sequence of the type strain whenever possible (Mora-Ruiz Mdel et al., 2015; Vidal et al., 2015). In general, one OPU can be equalized to prokaryotic species due to the low internal identity variability. Identical or nearly identical sequences (>98.7% identity) with type strain sequences were defined as known species. When the OPU represented an independent lineage within a clear genus, it was defined as a potentially new species. When the affiliation of an OPU was unclear but closely affiliated with known genera, within families or higher taxa, it was assigned as potential new “higher taxon.” When an OPU had only closest relatives that were represented by uncultured organisms, and did not affiliate within any classified taxon, it was designated as an “uncultured taxa” category.

### Fine-Tuned Phylogenetic Reconstruction of Selected OPUs

To better understand the taxonomic position of the selected OPUs representing new “higher taxa,” a maximum-likelihood (ML) reconstruction was performed. For each selected OPU, different sequence datasets were selected from the LTP123 database. The alignments of the OPUs sequence representatives were manually improved in relation to the reference sequences, and *de novo* phylogenetic trees were reconstructed using RAxML as previously described (Munoz et al., 2016). Node stability was evaluated using a rapid bootstrapping analysis (RAxML, 100 runs).

### Statistical Analysis

Rarefaction curves were calculated based on OPU abundances using PAST v3.18 software^3^ (Hammer Ø. et al., 2001). The community structure in relation to the sampling location and inter-individual variability of OPUs was statistically analyzed using non-metric multidimensional scaling (NMDS) as described previously (Mora-Ruiz Mdel et al., 2016). A NMDS stress value less than 0.396, which represented an acceptable value for the 104 samples (Kenneth Sturrock, 2000), was evidence of the goodness of the analysis. The divergence between samples based on the OPU abundances and compositions was performed by using the non-parametric Kruskal–Wallis tests, as the data did not meet the assumptions of normality and homoscedasticity. Additionally, to test the average contribution of each OPU to sample segregation, similarity percentage (SIMPER) analysis was performed. This analysis was carried out using the Vegan package in rv3.4.1.^4^ (Oksanen, 2018).

## Results

### Species Level Fecal Microbiota Composition by OPUs Analysis

**FIGURE 1:**
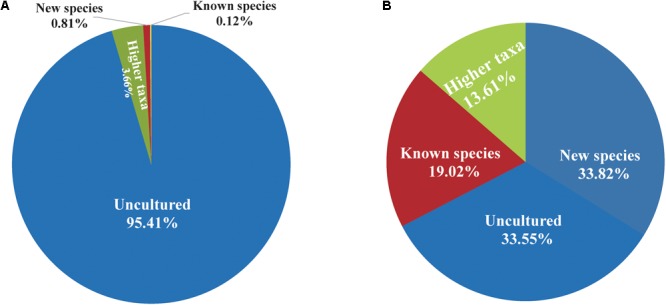
Proportion of uncultured bacteria in the feces of Tibetan antelope. **(A)** The proportion of reads. The number of reads classified into uncultured bacteria, bacteria at higher taxa levels, new species, and known species was 827,656, 31,743, 6,970, and 1,065, respectively. **(B)** The proportion of OPUs. The number of OPUs that classified into uncultured bacteria, higher taxa levels, new species and known species was 254, 103, 256, and 144, respectively.

The PacBio platform rendered a total of 1,254,752 raw 16S rRNA sequence reads for the 104 fecal samples of the animal, with an average of 12,065 ± 4,117 reads per sample. After quality filtering and removal of chimeras, a total of 867,434 (69.1%) high-quality reads of the 16S rRNA amplicons were obtained, with an average 8,341 ± 2,811 reads per sample, ranging from 3,697 to 16,519 among individuals. The sequences were on average 1,444 ± 2.21 bp in length (**Supplementary Table S1**). Reads of high quality were clustered into 61,258 unique OTUs at an identity threshold of 98.7%. The representative sequences of each OTU affiliated with 757 OPUs in total. We devised four different categories (**Figure 1**) as follows: (i) uncultured (254 OPUs; 827,656 sequences), that comprised sequences not affiliated with any known family and all closest relatives were unclassified lineages of existing orders or classes; (ii) higher level taxa (103 OPUs; 31,743 sequences) comprising sequences that formed single lineages that would represent new genera or families within known orders; (iii) potentially new species (256 OPUs; 6,970 sequences) belonging to known genera; and (iv) known species (144 OPUs; 1,065 sequences). Rarefaction analysis mostly showed saturation (**Supplementary Figures S1B,C**). The error rates generated by PacBio sequencing ranged from 0.09 to 0.13% for each sample (**Supplementary Figure S1A**), which was in accordance with our previous study (Meng et al., 2017). Three OPUs, namely, OPU 726 assigned as uncultured *Mogibacterium* sp. with 35 representative sequences, OPU 737 assigned as *Anaerofustis* sp. with 96 representative sequences and OPU 745 assigned as *Romboutsia* sp. with 27 representative sequences were selected for a deeper analysis of insertion and deletion rates, by comparing the alignments with the corresponding reference sequences. The insertion and deletion rates were 0.3% ± 0.32 and 2.75% ± 1.65, respectively (**Supplementary Table S2**), which were comparable to the rates observed in vultures. However, we are confident with the accuracy of our treeing approaches as we always used a 30% conservational filter implemented in the LTP123 database (Yarza et al., 2010), which would minimize the effects of insertions. In addition, as we reconstructed the *de novo* tree topologies using the neighbor joining algorithm and almost full-length gene sequences, the tree topologies gained in reliability due to the larger information content, noise removal by using conservational filters, and not sticking to a stable and non-modifiable topology that would result from the use of the parsimony tool adequate for partial sequences (Ludwig et al., 1998; Yarza et al., 2014).

### The Samples Are Biological Replicates

Distinct sample compositions were used to generate a NMDS plot (**Supplementary Figure S2**) to evaluate whether the samples were biological replicates and to determine which sample contributed to the dissimilarities. The plot clearly showed that all samples, except for the five outliners (TA30, TA50, TA51, TA59, and TA393), were nearly identical as indicated by the Kruskal–Wallis test (**Supplementary Table S3**) with a *p*-value higher than 0.05 among all samples except the five outliers. This significance value is indicated by the circle embracing all replicates in the NMDS plot (**Supplementary Figure S2**). In addition, we generated a SIMPER analysis (**Supplementary Table S4**) to evaluate which OPUs contributed to the segregation of the five outliers from the majority of samples (99 samples). Remarkably, the five outliers were those showing higher diversity trends (**Supplementary Figure S1C**), but we could not find any exclusive OPU for these samples. The differences were mostly due to the distinct occurrence of common OPUs, which we could discard due to sample contamination. Despite the irrelevant differences of the five outliers, we considered all 99 remaining samples as biological replicates, with no biogeographical segregation trend, nor location specificity.

### The Precision in the Determination of the Fecal Microbiota Composition

**FIGURE 2:**
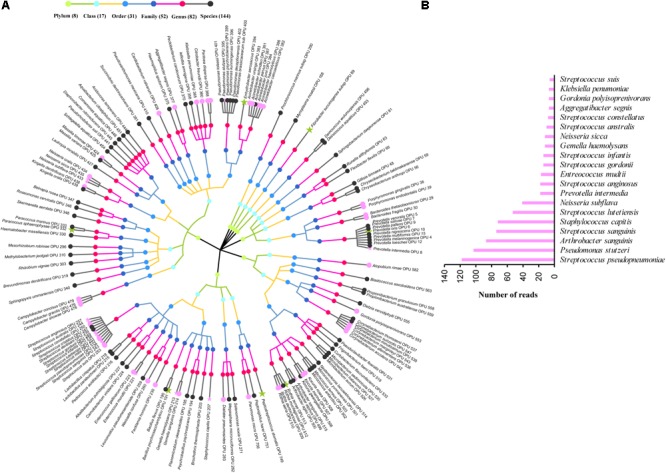
Profiles of known species. **(A)** Taxonomic tree of 144 known species. Each dot represents a taxonomic entity. From the inner to the outer circles, the taxonomic levels range from phylum to species. Dots of different colors indicate different taxonomic levels according to the color key shown. Numbers in parentheses indicate the total number of unique taxonomies detected at each level. The most abundant species, which were composed of more than 10 reads are denoted by asterisk. Species in pink indicate those having medical significance, among which the top 20 species, which were composed of more than 10 reads are denoted by pink asterisk. **(B)** Sequences numbers of the top 20 known species having medical significance.

**FIGURE 3:**
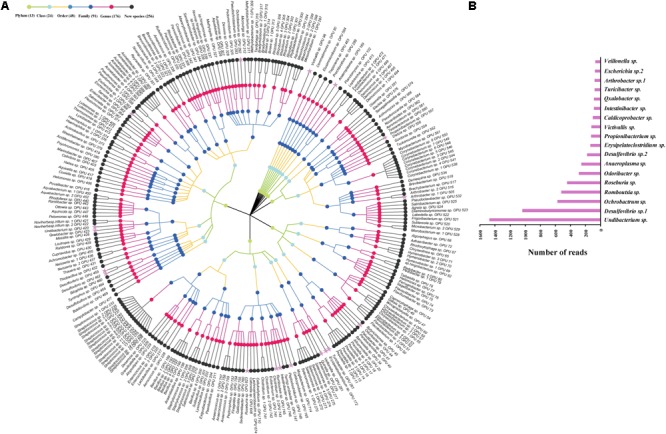
Profiles of new species. **(A)** Taxonomic tree of 256 new species. The most abundant species, which were composed of more than 50 reads are showed by pink asterisk. **(B)** The top 18 abundant new species with more than 50 reads.

**FIGURE 4:**
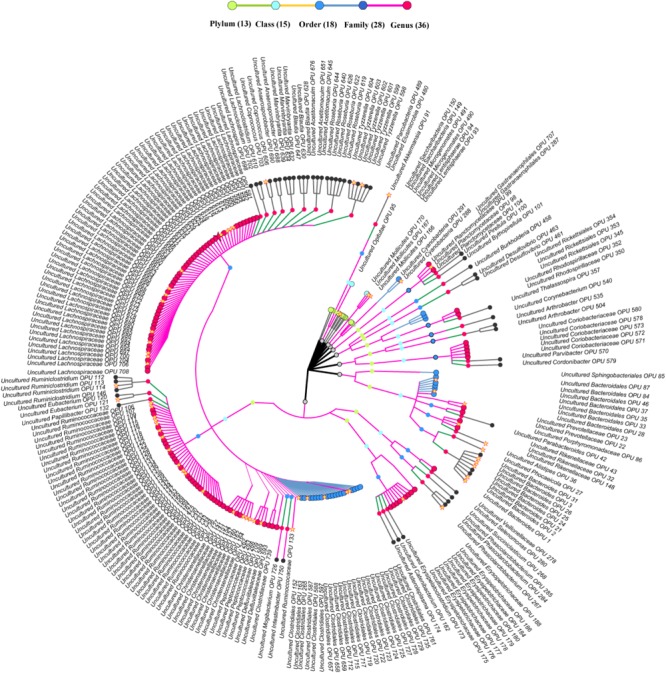
Taxonomic tree of uncultured bacteria. Each dot represents a taxonomic entity. From the inner to the outer circles, the taxonomic levels range from phylum to genus. Dots of different colors indicate different taxonomic levels according to the color key shown. The outermost taxa of each OPU show the highest taxa level it can reach. Numbers in parentheses indicate the total number of unique taxonomies detected at each level. The core OPUs shared by all antelopes are showed by asterisk.

The entire fecal microbiome affiliated with 24 phyla, 38 classes, 67 orders, 122 families, and 252 genera in total (**Supplementary Figure S3** and **Supplementary Table S5**). Of the 757 OPUs, 144 (19.02%) affiliated with known species (>98.7% identity with their respective type strain), which belonged to 8 phyla, 17 classes, 31 orders, 52 families, and 82 genera (**Figure 2**). Notably, the sequences of the 144 bacterial species occupied only 0.12% of total reads (**Figure 1**), and their abundances in each individual animal were also low, with less than 0.51% reads for a single animal. In addition, 55 out of these known species, affiliated with medically significant bacteria that had been reported as associated with human or animal diseases (**Figure 2A**). Their abundances were low, with sequence numbers ranging from 1 to 118 among individual samples. Among the 55 known species of medical significance, only 20 comprised of more than 10 reads (**Figure 2B**). A total of 256 OPUs were classified as potential new species affiliating with 176 classified genera (**Figure 3**), but representing independent lineages within each respective genus. These 256 potentially new species accounted for 0.8% of the total reads, which varied from 0 to 1.3% among individual samples. The most abundant potentially new species was *Undibacterium* sp., consisting of 1,476 reads. There were 17 potentially new species comprising of at least 50 reads (**Figure 3**). A total of 103 OPUs were assigned as potentially higher-level taxa, which were very likely to represent new genera, families or higher taxa. These comprised 3.66% of the total reads. Three of them, namely, OPU 124 (*Ruminococcaceae*_family category), OPU 130 (*Ruminococcaceae*_family category), and OPU 128 (*Ruminiclostridium*_genus category), were shared by all 104 fecal samples. On the other hand, 254 out of the 757 OPUs were classified as uncultured bacteria (**Figure 4**), as their closest relatives were reference sequences from environmental surveys, and they did not affiliate with any clearly classified family or higher taxa. Remarkably, the sequences of uncultured bacteria accounted for 95.41% of the total sequences (827,656 reads) (**Figure 1A**).

### The Uncultured Core Fecal Microbiome

**FIGURE 5:**
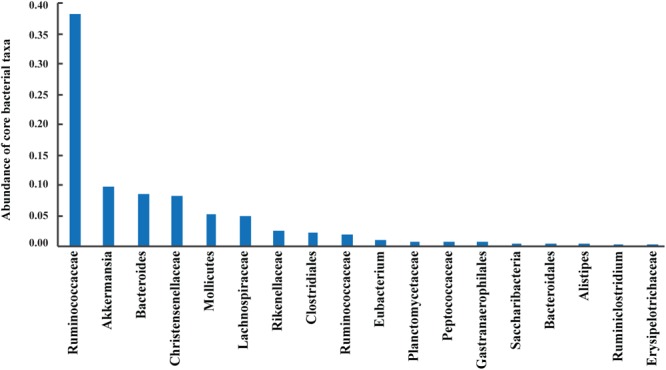
Abundances of core bacterial taxa shared by all samples. Sequences numbers of these predominant taxa from left to right were 329,996, 85,042, 73,443, 72,199, 44,772, 43,430, 21,233, 20,151, 17,182, 9,182, 7,041, 6,865, 6,084, 4,630, 4,135, 4,050, 2,302, and 1,576, respectively.

A total of 42 out of 757 OPUs were shared by all fecal samples, accounting for 86.84% (753,313 reads) of the total reads, ranging from 0.13 to 13.62% for each OPU among individual samples (**Table 2** and **Supplementary Table S6**). The most predominant core bacteria were uncultured *Ruminococcaceae*, which comprised 38% of the total reads, followed by uncultured *Akkermansia* (9.8%), uncultured *Bacteroides* (8.5%), uncultured *Christensenellaceae* (8.3%) (**Figure 5**). These OPUs, which comprised approximately 65% of the entire microbiome, belonged to strict anaerobic lineages, as is typical of feces (Nava and Stappenbeck, 2011; Goodrich et al., 2014). Remarkably, none of the 42 members of the core fecal microbiome was a known species, three of them were assigned as potentially new genera within the family *Ruminococcaceae*, and the remaining 39 members were considered as uncultured taxa (**Table 2**).

**Table 2:** List of core fecal taxa and their abundance.

Core taxa		Reads number	OPUs included
Phylum level	Uncultured *Saccharibacteria*	4,630	OPU 149
Class level	Uncultured *Mollicutes*	44,772	OPU 166
Order level	Uncultured *Clostridiales*	20,151	OPU 587, OPU 712, OPU 725, OPU 727
	Uncultured *Gastranaerophilales*	6,084	OPU 287
	Uncultured *Bacteroidales*	4,135	OPU 33
Family level	Uncultured *Ruminococcaceae*	329,996	OPU 106, OPU 107, OPU 115, OPU 116, OPU 117,
			OPU 123, OPU 131, OPU 139, OPU 142, OPU 144
	Uncultured *Christensenellaceae*	72,199	OPU 151, OPU 160, OPU 164
	Uncultured *Lachnospiraceae*	43,430	OPU 612, OPU 617, OPU 618, OPU 664, OPU 666
			OPU 668, OPU 704
	Uncultured *Rikenellaceae*	21,233	OPU 32
	*Ruminococcaceae*	17,182	OPU 124, OPU 130
	Uncultured *Planctomycetaceae*	7,041	OPU 98
	Uncultured *Peptococcaceae*	6,865	OPU 262, OPU 266
	Uncultured *Erysipelotrichaceae*	1,576	OPU 179
Genus level	Uncultured *Akkermansia*	85,042	OPU 91
	Uncultured *Bacteroides*	73,443	OPU 1, OPU 2, OPU 25
	Uncultured *Eubacterium*	9,182	OPU 121
	Uncultured *Alistipes*	4,050	OPU 36
	*Ruminiclostridium*	2,302	OPU 108
Total		753,313	42

### Identification of Novel Bacterial Candidate at High Taxa Levels

**FIGURE 6:**
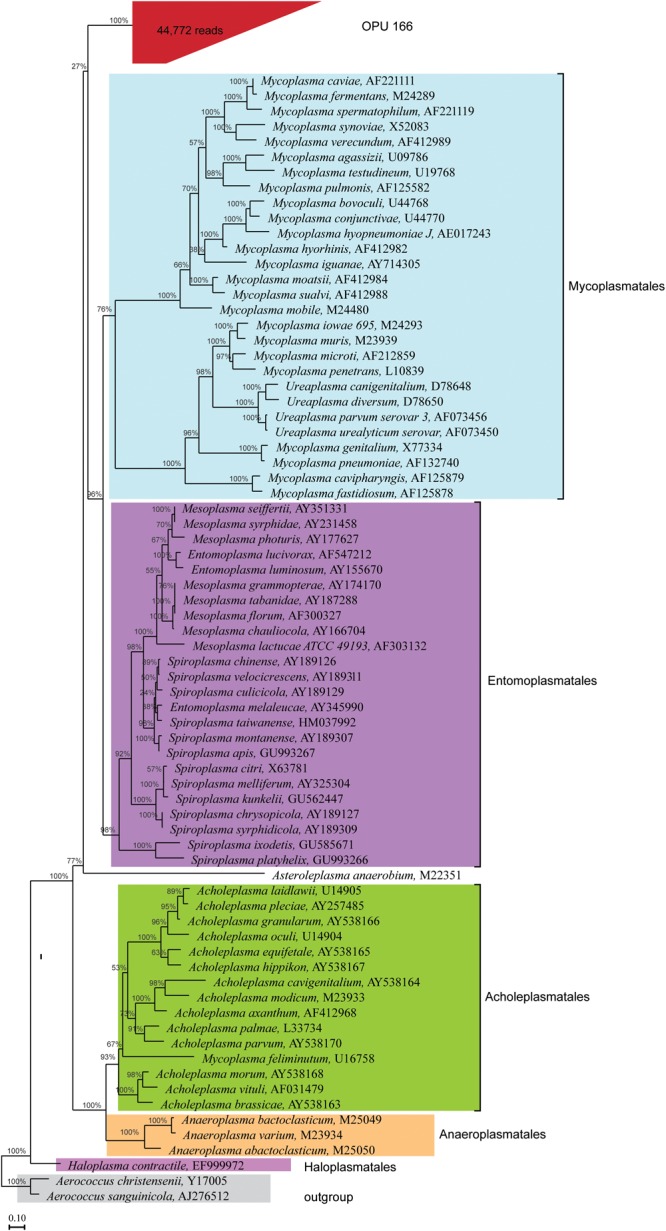
Phylogeny of core uncultured *Mollicutes*. A phylogenetic comparison was carried out using the representatives 16S rRNA sequences of OPU 166, which was defined as uncultured *Mollicutes*, type strains of five orders identified in the *Mollicutes* class, and two type strains in *Firmicutes* phylum, which were used as outgroup. OPU 166 is grouped in red box with the total number of reads shown in parentheses. The maximum likelihood phylogeny was constructed using RAxML with 100 replications (*Escherichia coli*-wide tree) as described in the Section “Materials and Methods.”

The uncultured OPU 166 with 44,772 reads affiliated as an independent branch within the *Mollicutes* class, possibly representing a new order different from the five classified orders (**Figure 6**) as it could be deduced from the lineage uniqueness and the sequence identity with the closest relative orders (Yarza et al., 2014). The representative sequences of OPU 166 showed an average identity of only 83% to LTP123 16S rRNA genes, with a minimum identity of 72.1% to *Mycoplasma penetrans* (L10839) and a maximum of 85.5% with *Entomoplasma melaleucae* (AY345990). Thus, OPU 166 may represent a novel bacterial candidate order within the *Mollicutes* class.

**FIGURE 7:**
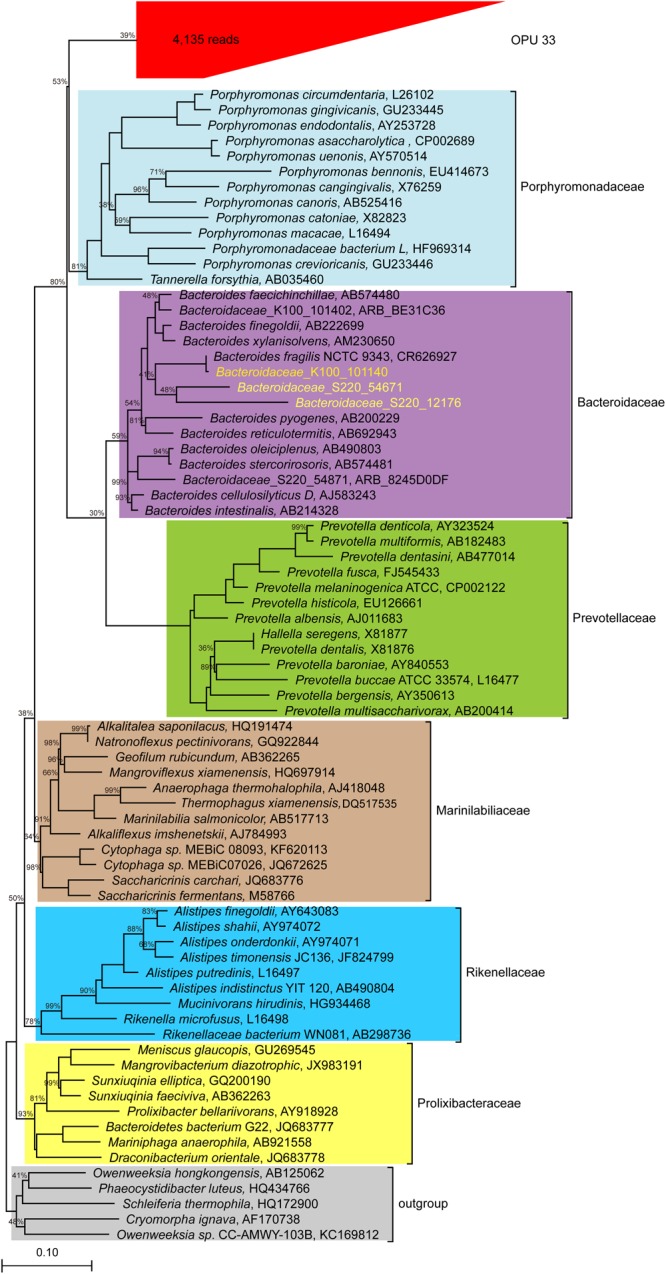
Phylogeny of core uncultured *Bacteroidale.* A phylogenetic comparison was carried out using the representative sequences of OPU 33 and type strains of six families identified in the *Bacteroidale* order. OPU 33 was grouped in the red box with the total number of reads shown in parentheses. The 16S rRNA sequences of *Bacteroidaceae* from human were downloaded and included in the analysis (http://metagenomics.anl.gov/linkin.cgi?project=17761) to assess reliability of the method we used, which are showed in yellow. The Maximum likelihood phylogeny was constructed using RAxML with 100 replications.

OPU 33 with 4,135 reads was assigned as uncultured *Bacteroidales*, and formed a distinct branch within the *Bacteroidales* order (**Figure 7**), showing a maximum identity of 86.8% to *Tannerella forsythia* (AB035460). Our data indicated that OPU 33 might represent a new bacterial candidate family within the order *Bacteroidales*.

OPU 151, OPU 160, and OPU 164 with 72,199 reads were assigned as uncultured *Christensenellaceae*. The family *Christensenellaceae* has only one genus, *Christensenella*, with only one species *Christensenella minuta*. Phylogenetic analysis showed OPU 164 affiliated with the same branch as the *Christensenella* genus, with a maximum identity of 92%, whereas OPU 160 and OPU 164 were phylogenetically distinct from the *Christensenella* genus. Thus, OPU 151, OPU 160 may constitute novel genera within the *Christensenellaceae* family (**Supplementary Figure S4A**).

We analyzed the shared core taxa, namely, *Ruminococcaceae, Lachnospiraceae, Akkermansia*, and *Christensenellaceae* between antelope and human, which were associated with human health or longevity. The 16s rRNA sequences of the four shared taxa from human were downloaded^5^ (Biagi et al., 2016) and compared with those from antelope. Despite the fact that 16s rRNA sequences from the human microbiome were 500 bp shorter on average, the OPU approach yielded relatively accurate results (Vidal et al., 2015). Phylogenetic analyses showed that the 16S rRNA sequences of the human *Christensenellaceae* family clustered in the same lineage with OPU 160 (**Supplementary Figure S4B**), but with a maximum identity of 92.6%, indicating that they might be a different species. In addition, OPU 617 and OPU 612 were placed in the same lineage with core *Lachnospiraceae* from human (Biagi et al., 2016) (**Supplementary Figure S5**), with a maximum identity of 93 and 95% with human *Lachnospiraceae* 16S rRNA sequences, respectively. These results suggested that they may respond to different specific lineages. Furthermore, two taxa designed as *Ruminococcaceae* and uncultured *Akkermansia* were shared with human and associated with either health or longevity based on the phylogenetic analysis (**Supplementary Figures S6, S7**). Our data indicated that the 16s rRNA sequences from human and antelope were not identical, but phylogenetically very close.

## Discussion

Next generation sequencing (NGS) and analysis of the 16S rRNA gene amplicons have revolutionized culture-independent microbial diversity studies research as they have allowed a significant increase in the sample size. However, a reliable identification of the sequences within the hierarchical taxonomic system requires 16S rRNA gene sequences covering almost full-length genes (Yarza et al., 2014). This premise is not achieved in current high-throughput 16S rRNA surveys, which mainly use Illumina or Roche 454 platforms. Our previously applied metataxonomic methodology, which was based on almost full-length amplicon sequencing by using the PacBio Technology, has allowed a fine-tuned diversity survey on Tibetan vultures (Meng et al., 2017). In the current study, we have generated a total of 867,434 high-quality full-length 16s rRNA sequences for 104 Tibetan antelope fecal samples from four different herds inhabiting in the Hoh Xil National Nature Reserve in the Qinghai Province. Remarkably 99 out of 104 sequences were treated as biological replicates, thus reinforcing the relevance of the results. The sequences were phylogenetically grouped into 757 OPUs, and according to the established definition and the internal variability, most of them could be considered as single species (Mora-Ruiz Mdel et al., 2016). The most remarkable results in our survey were that we could only assign approximately 19% of the OPUs to a classified species using a conservative identity threshold of 98.7% (Stackebrandt and Ebers, 2006). Furthermore, 55 OPUs were considered as medically significant bacteria, and the sum of their overall abundances was less than 0.5%. To the best of our knowledge, this is the first report of Tibetan antelope serving as an animal host or a reservoir for pathogens such as *Klebsiella pneumoniae, Streptococcus suis, S. sanguinis, S. lutetiensis, S. anginosus*, and *S. infantis*. In addition to the known species, 34% of the OPUs were considered as potentially new species within known genera, while accounting for a very low percentage (0.8%) of the total reads. A low percentage of the OPUs (13%) represented putative high taxa (new genera or families), which accounted for approximately 3.65% of the total reads. Thus, it was remarkable that 34% of the OPUs could not be assigned to any genus or family using the conservative thresholds of Yarza et al. (2014), belonged to phylogenetic branches mostly formed by environmental sequences and these accounted for the great majority (95.41%) of the total reads. Altogether, the percentage of OPUs identified at the genus or species level was approximately 64%, an amount comparable to the 70% observed for human microbiomes using the OPU approach (Vidal et al., 2015). The most remarkable finding, however, was that although the OPU richness was similarly distributed between clear and unclear classifications, the abundances were strongly balanced for those yet to be described microbial diversity.

When comparing our microbiota results with those of other ruminants, such as sheep, lambs, and cattle, we found a general predominance of *Firmicutes* and *Bacteroidetes*, with *Ruminococcaceae* and *Lachnospiraceae* being the most representative microbial families of the former phyla (Kim et al., 2014; Zeng et al., 2015; Tanca et al., 2017), which was in accordance with our results obtained in the antelope. Consistent with other studies, numerous sequences could not be assigned to a known genus, and the most dominant unclassified group was unclassified *Ruminococcaceae*, which could be classified to a family but not to a genus (Kim et al., 2014). A previous study on the composition and quantification of the microbial community in the gastrointestinal tract in Chinese Mongolian sheep by PCR-DGGE and real-time PCR, revealed a predominance of *Firmicutes* and *Bacteroidetes*, with *Ruminococcus flavefaciens* showing a significant difference in their abundance (Zeng et al., 2015). Despite the different approaches used in these studies, comparisons between the microbiota in the ruminants indicated the predominance of similar bacteria, which were mostly involved in catabolism (Tanca et al., 2017).

As expected, the fecal core microbiome of herbivorous Tibetan antelopes and carnivorous vultures, which resides in the same geographical environment were totally different. We have analyzed the fecal microbiome of old world vultures in the same area using the same strategy and methods (Meng et al., 2017). The vultures are the most successful scavengers, feeding on the carcasses of dead animals without suffering any apparent illness. We found that only 6 out of 314 OPUs were shared by all 9 old world vultures, including *Clostridium perfringens, Peptostreptococcus russellii, Eubacterium moniliforme/Eubacterium multiforme, Peptostreptococcus vulture* sp. and *Sporacetigenium vulture* sp. While none of them was a member of the fecal core microbiome of the Tibetan antelope (Meng et al., 2017). Interestingly, we found that the Tibetan antelope and human shared several bacterial taxa. It was reported that the fecal microbiota in all age groups of humans was dominated by three families, namely, *Bacteroidaceae, Lachnospiraceae*, and *Ruminococcaceae* (Biagi et al., 2016). Two of these families were detected in Tibetan antelopes as fecal core taxa. Furthermore, three families, namely, *Akkermansia, Bifidobacterium*, and *Christensenellaceae* were considered as to be associated with longevity in humans. Their abundance increased with aging of people with longevity (Biagi et al., 2016). Interestingly, *Akkermansia* and *Christensenellaceae* were members of the fecal core microbiome of Tibetan antelopes. Those findings suggest that vegetarian diet, which may include caloric restrictions and vitamins, might has been one of the factors that had a positive impact on longevity. It should be mentioned that Tibetan antelopes are herbivores and feed on mostly grass.

The *Ruminococcaceae* family, the most predominant taxon identified in this study, includes the anaerobic species that primarily inhabit in the caecum and the colon (Donaldson et al., 2016). As a member of the short chain fatty acid (SCFA) producers, *Ruminococcaceae* has been implicated in the degradation of diverse polysaccharides and fibers (Hooda et al., 2012). *Akkermansia muciniphila* is present in approximately 3% of the adult colon, rendering it one of the most abundant and beneficial intestinal species and testifying its apparent safety. It was depleted in fecal samples from adults suffering from a variety of diseases, including obesity, metabolic syndrome and diabetes. Interestingly, the uncultured *Akkermansia* (OPU 91) was detected in all antelope fecal samples and accounted for 9.8% of total reads. *Bacteroides* is a genus of obligate anaerobic bacteria. It comprises the most substantial portion of the mammalian gastrointestinal microbiota, and plays a fundamental role in the processing of complex molecules to simpler ones in the host’s intestine. One remarkable finding in this study was that uncultured bacteria phylogenetically classified as *Lachnospiraceae, Bacteroidales, Clostridiales*, and *Ruminococcaceae* in this study may play critical roles in ruminal biohydrogenation (Huws et al., 2011). Taken collectively, these results reveal that the fecal core microbiome of the antelope is formed by uncultured bacteria that may have beneficial roles in the promotion of health and anti-intestinal dysbiosis. Although the health benefits of dietary fiber have long been suggested, the direct effects of fiber polysaccharides on the composition and physiology of the microbiota has remained elusive. Thus, the relationship among the gut microbiota, diet, and intestinal barrier dysfunction should be exploited for dietary therapeutics in the future.

## Conclusion

By using the metataxonomic approach based on OPU and almost full-length PacBio amplicon sequences, we provided at the first time, at the species level, the taxonomic structure of the fecal microbiome of the Tibetan antelopes that live in the highly elevated and depopulated Qinghai-Tibet Plateau with a low partial pressure of oxygen and a high level of ultraviolet radiation. Remarkably, we found that the predominant and core bacterial taxa of the antelope fecal microbiome were unknown species, and several may represent potentially new genera within the corresponding families. In addition, 144 OPUs were classified as known species, including 55 pathogenic bacteria, such as *Klebsiella penumoniae, S. suis, S. sanguinis, S. lutetiensis, S. anginosus*, and *S. infantis*. Therefore, the Tibetan antelope might be considered as an animal reservoir for those pathogens.

## Availability of Data and Materials

All data generated or analyzed during this study are included in this published article, as well as its Supplementary Material files.

## Author Contributions

XB, SL, JY, DJ, JP, and YX collected samples and performed laboratory assays. XB, SDM, RR-M, and JX analyzed the data and wrote the manuscript. RR-M and JX contributed resources. JX designed the project.

## Conflict of Interest Statement

The authors declare that the research was conducted in the absence of any commercial or financial relationships that could be construed as a potential conflict of interest.

## Footnotes

1 www.pacb.com/devnet/

2 http://folk.uio.no/ohammer/past/

3 http://palaeo-electronica.org/2001_1/past/issue1_01.htm

4 https://cran.r-project.org/web/packages/vegan/vignettes/intro-vegan.pdf

5 http://metagenomics.anl.gov/linkin.cgi?project=17761

## Abbreviations

LTP: Living Tree Project
OPU: operational phylogenetic unit
OTU: operational taxonomic unit
RDP: Ribosomal Database Project
SINA: SILVA Incremental Aligner
