# Supplementary material for: Precise Fecal Microbiome of the Herbivorous Tibetan Antelope Inhabiting High-Altitude Alpine Plateau
Source: Front Microbiol. 2018 Sep 28;9:2321. doi: 10.3389/fmicb.2018.02321 (PMC6172336; doi:10.3389/fmicb.2018.02321)
Supplement: Supplementary file 1 [file Data_Sheet_1.PDF]

Supplementary Material

Precise Fecal Microbiome of The Herbivorous Tibetan Antelope Inhabiting High-altitute Alpine Plateau

Xiangning Bai, Shan Lu, Jing Yang, Dong Jin, Ji Pu, Sara Díaz Moyá, Yanwen Xiong, Ramon Rossello-Mora and Jianguo Xu*

***Correspondence:** Jianguo Xu: xujianguo@icdc.cn

Supplementary Tables

Table S1 Quality estimation of the 16S rRNA sequencing by PacBio

Sample	Raw reads	Final reads[a]	Length (bp)	Q20(%)	Q30(%)	Extract effective[b]
TA10	12937	9827	1446	98.98	98.02	0.76
TA104	18906	12982	1446	99.16	98.17	0.69
TA106	15624	11485	1443	99.02	98.05	0.74
TA11	9175	6389	1444	98.96	98.02	0.70
TA111	11372	8668	1439	98.97	98.03	0.76
TA112	20806	12537	1446	99.12	98.11	0.60
TA114	16516	9329	1445	99.14	98.18	0.56
TA115	13078	9788	1444	98.99	98.05	0.75
TA116	13043	7499	1446	99.14	98.17	0.57
TA117	10586	7102	1442	98.93	97.97	0.67
TA118	11106	7513	1442	98.95	98.01	0.68
TA119	22681	15425	1446	99.16	98.18	0.68
TA120	11849	6071	1445	99.1	98.12	0.51
TA121	13691	10059	1445	98.94	97.96	0.73
TA122	11341	8406	1446	99.00	98.06	0.74
TA123	17856	9377	1444	99.12	98.13	0.53
TA124	11919	9223	1445	99.02	98.11	0.77
TA125	7501	4983	1441	98.97	97.99	0.66
TA126	10125	6123	1444	98.98	98.01	0.60
TA127	13991	9843	1446	99.02	98.05	0.70
TA13	10108	6657	1443	98.98	98.02	0.66
TA130	8943	5779	1443	98.97	98.01	0.65
TA132	8266	6238	1443	99.02	98.07	0.75
TA133	7663	4970	1442	99.00	98.06	0.65
TA136	13347	9747	1439	99.00	98.02	0.73
TA137	9790	6130	1444	98.99	98.02	0.63
TA141	10957	8308	1445	98.99	98.03	0.76
TA142	16971	10293	1447	99.13	98.14	0.61
TA145	10420	7516	1442	99.02	98.08	0.72
TA146	10484	7567	1445	99.01	98.04	0.72
TA148	13476	7181	1446	99.13	98.16	0.53
TA150	8074	5618	1446	99.03	98.09	0.70
TA151	17400	12173	1445	98.98	98.02	0.70
TA155	6949	4704	1440	99.17	98.24	0.68
TA16	11851	7685	1443	99.13	98.12	0.65
TA160	6934	4377	1446	99.00	98.03	0.63
TA161	10975	7417	1446	99.00	98.05	0.68
TA163	12083	9023	1443	98.97	98.01	0.75

Sample	Raw reads	Final reads[a]	Length (bp)	Q20(%)	Q30(%)	Extract effective[b]
TA164	11738	8649	1448	99.01	98.04	0.74
TA165	5996	4439	1447	99.05	98.10	0.74
TA166	12543	9254	1446	99.04	98.11	0.74
TA168	13762	10404	1446	98.98	98.01	0.76
TA169	9695	6551	1446	98.99	98.02	0.68
TA172	8369	5638	1446	99.02	98.07	0.67
TA175	6440	4432	1446	99.01	98.05	0.69
TA177	8856	5811	1442	99.15	98.20	0.66
TA178	10141	7422	1447	99.02	98.06	0.73
TA2	12356	9478	1442	98.98	98.03	0.77
TA23	9976	6303	1446	98.99	98.01	0.63
TA25	14266	10144	1436	98.94	97.98	0.71
TA27	7658	5195	1440	98.99	98.02	0.68
TA28	24152	16519	1447	99.18	98.21	0.68
TA29	22939	15940	1440	99.00	98.05	0.69
TA30	12094	9018	1445	98.95	97.95	0.75
TA36	12471	9527	1447	98.97	98.01	0.76
TA361	8808	5452	1446	99.00	98.04	0.62
TA362	7478	4840	1447	99.04	98.12	0.65
TA366	6362	4717	1443	99.00	98.06	0.74
TA369	11127	8356	1446	99.01	98.06	0.75
TA37	17897	13430	1446	99.04	98.09	0.75
TA373	10715	8269	1444	98.97	98.02	0.77
TA375	14030	10633	1444	98.97	98.01	0.76
TA379	11046	8464	1445	98.97	98.01	0.77
TA38	22434	14749	1445	99.15	98.19	0.66
TA380	12240	9376	1442	98.98	98.03	0.77
TA381	8296	6068	1442	98.96	98.06	0.73
TA384	9770	7610	1444	98.94	98.01	0.78
TA389	6751	5267	1444	98.95	98.03	0.78
TA39	13491	8997	1447	99.18	98.24	0.67
TA390	7619	5937	1443	98.96	98.05	0.78
TA393	7166	5634	1441	98.96	98.04	0.79
TA394	6257	4569	1444	98.93	98.02	0.73
TA395	6428	3746	1446	99.17	98.24	0.58
TA396	6589	5236	1444	98.94	98.03	0.79
TA40	13190	8712	1446	99.16	98.20	0.66
TA42	10655	6435	1443	99.12	98.14	0.60
TA43	11909	7468	1443	99.13	98.16	0.63
TA44	13547	9875	1445	99.04	98.09	0.73
TA45	20834	14347	1447	99.15	98.16	0.69
TA48	13558	9015	1442	98.99	98.03	0.66
TA49	13808	9452	1443	98.98	98.02	0.68

Sample	Raw reads	Final reads[a]	Length (bp)	Q20(%)	Q30(%)	Extract effective[b]
TA5	5484	3697	1444	99.01	98.05	0.67
TA50	16455	12090	1443	99.01	98.05	0.73
TA51	12946	9275	1440	99.00	98.05	0.72
TA53	18115	10606	1445	99.13	98.14	0.59
TA55	10614	7097	1442	98.98	98.00	0.67
TA59	11589	7779	1443	98.98	98.00	0.67
TA6	10968	7659	1440	98.98	98.00	0.70
TA61	19403	13192	1441	99.00	98.05	0.68
TA62	15736	11063	1443	98.99	98.02	0.70
TA68	15705	11731	1442	99.00	98.03	0.75
TA69	12066	8825	1443	99.01	98.06	0.73
TA7	14929	10821	1443	99.03	98.08	0.72
TA8	11432	8740	1441	98.93	97.92	0.76
TA83	11044	8104	1445	98.97	98.01	0.73
TA84	6327	4657	1444	99.02	98.06	0.74
TA86	17093	12745	1444	99.05	98.11	0.75
TA87	11294	8465	1446	98.96	97.99	0.75
TA88	12947	8161	1443	99.13	98.15	0.63
TA89	12541	9424	1440	99.01	98.05	0.75
TA9	17358	10621	1445	99.13	98.15	0.61
TA90	10195	6516	1445	99.15	98.17	0.64
TA96	6047	3723	1443	98.97	98.01	0.62
TA97	10213	7053	1444	98.98	97.99	0.69
Total	1,254,752	867,434	150,171	-	-	72.00
Average	12064.92	8340.71	1443.95	-	-	0.69
SD	4116.98	2810.53	2.21	-	-	0.06

[a] The number of high quality reads for analysis
[b] The proportion of high quality reads for analysis in raw reads.

Table S2 The rates of insertion and deletion of three OPUs from PacBio

OPU	No. Seq	Delet/Seq*	SD	Insert/Seq*	SD	Delet%	SD	Insert%	SD
OPU 726	35	69.89	13.24	0	0	4.90	0.97	0	0
OPU 737	96	33.05	17.16	6.50	6.50	2.35	1.25	0.46	0.24
OPU 745	27	19.48	12.45	1.70	5.91	1.39	0.90	0.12	0.41
All	158	38.89	4.24	4.57	13.81	2.75	1.65	0.30	0.32

*: mean of inserted or deleted nucleotides in each sequence

SD: standard deviation

Table S3 Kruskal-wallis results

Sample	H*	p-value
TA30	14.337	0
TA50	12.787	0.00087
TA51	14.002	0.00090
TA59	11.822	0
TA393	10.999	0.00107

* critical chi-square value and p-value for the comparisons between the 5 outliers (TA30, TA50, TA51, TA59 and TA393) and the rest of samples that constitutes biological replicates.

Table S4 Similarity percentage (SIMPER) analysis based on OPU abundances between 5 outliers and samples that constitute biological replicates.

A:

TA30	Average	St. Dev	p-value
OPU 33	0.01134000	0.00314300	0.001
OPU 557	0.00095920	0.00020230	0.001
OPU 471	0.00058830	0.00009726	0.001
OPU 9	0.00041400	0.00006479	0.001
OPU 160	0.03030000	0.00718900	0.002
OPU 21	0.00767100	0.00177100	0.002
OPU 93	0.00465000	0.00095390	0.002
OPU 292	0.00203700	0.00064480	0.002
OPU 749	0.00065060	0.00010180	0.002
OPU 580	0.00061910	0.00011800	0.002
OPU 275	0.00059150	0.00009255	0.002
OPU 515	0.00043280	0.00012010	0.002
OPU 8	0.00040970	0.00007526	0.002
OPU 308	0.00039950	0.00007998	0.002
OPU 274	0.00029570	0.00004628	0.002
OPU 190	0.00029570	0.00004628	0.002
OPU 282	0.00029500	0.00004595	0.002
OPU 380	0.00023660	0.00003702	0.002
OPU 460	0.00022940	0.00004498	0.002
OPU 716	0.00043970	0.00010260	0.004
OPU 234	0.00110900	0.00018910	0.009
OPU 243	0.00082360	0.00013500	0.009
OPU 257	0.00029520	0.00004705	0.009
OPU 281	0.00070370	0.00011360	0.011
OPU 433	0.00047120	0.00007709	0.011
OPU 682	0.00042350	0.00012580	0.012
OPU 151	0.10330000	0.03221000	0.013
OPU 26	0.05192000	0.01494000	0.021
OPU 164	0.00208400	0.00088720	0.022
OPU 473	0.00034920	0.00006479	0.022
OPU 461	0.00028400	0.00010680	0.022
OPU 27	0.00301800	0.00098410	0.033
OPU 643	0.00025080	0.00008651	0.035
OPU 23	0.00306900	0.00119000	0.041
OPU 62	0.00022730	0.00005126	0.043
OPU 459	0.00206300	0.00071820	0.045
OPU 357	0.00082090	0.00031810	0.045

B:

TA50	Average	St. Dev	p-value
OPU 394	0.00019430	0.00003569	0.001
OPU 357	0.00167200	0.00038090	0.002
OPU 56	0.00019870	0.00002812	0.002
OPU 447	0.00044860	0.00005981	0.004
OPU 632	0.00026800	0.00007013	0.004
OPU 388	0.00024800	0.00003448	0.004
OPU 384	0.00019960	0.00002651	0.005
OPU 686	0.00336800	0.00093150	0.006
OPU 490	0.00033570	0.00007059	0.006
OPU 111	0.00198400	0.00049420	0.007
OPU 113	0.00085600	0.00024980	0.007
OPU 62	0.00039020	0.00006537	0.011
OPU 633	0.00026630	0.00006928	0.011
OPU 612	0.03484000	0.00977300	0.013
OPU 23	0.00393200	0.00107300	0.014
OPU 611	0.00103600	0.00024070	0.014
OPU 420	0.00255100	0.00074130	0.016
OPU 332	0.00019600	0.00002889	0.017
OPU 21	0.00433700	0.00108600	0.022
OPU 725	0.00140100	0.00054800	0.026
OPU 96	0.00055510	0.00012730	0.026
OPU 557	0.00041330	0.00010150	0.026
OPU 234	0.00068570	0.00010780	0.027
OPU 243	0.00049510	0.00007153	0.027
OPU 433	0.00019790	0.00003014	0.027
OPU 675	0.00019280	0.00004083	0.031
OPU 25	0.01629000	0.00599400	0.033
OPU 93	0.00192600	0.00048740	0.033
OPU 689	0.00043490	0.00013670	0.04
OPU 2	0.00955700	0.00363600	0.041
OPU 587	0.00657600	0.00252300	0.044
OPU 144	0.04125000	0.01786000	0.045
OPU 458	0.00070990	0.00026770	0.047
OPU 352	0.00036500	0.00014550	0.049

C:

TA51	Average	St. Dev	p-value
OPU 545	0.00029070	0.00004572	0.001
OPU 249	0.00029020	0.00004635	0.001
OPU 54	0.00028910	0.00004716	0.001
OPU 250	0.00017470	0.00002694	0.001
OPU 648	0.00017470	0.00002694	0.004
OPU 149	0.02115000	0.00450900	0.006

OPU 1	0.06865000	0.02270000	0.01
OPU 287	0.00871200	0.00319700	0.016
OPU 100	0.00299800	0.00048330	0.017
OPU 473	0.00045920	0.00008403	0.019
OPU 234	0.00109200	0.00018380	0.022
OPU 243	0.00081100	0.00013110	0.022
OPU 435	0.00017430	0.00002805	0.022
OPU 374	0.00023200	0.00003747	0.025
OPU 602	0.00017470	0.00007116	0.033
OPU 26	0.05089000	0.01458000	0.034
OPU 281	0.00034350	0.00005966	0.036
OPU 33	0.00575900	0.00192100	0.049
OPU 689	0.00034700	0.00013520	0.05

D:

TA59	Average	St. Dev	p-value
OPU 25	0.01355000	0.00656600	0.044
OPU 207	0.00068060	0.00014700	0.019
OPU 557	0.00059330	0.00015520	0.012
OPU 669	0.00039680	0.00019710	0.047
OPU 631	0.00025970	0.00009721	0.024
OPU 54	0.00025350	0.00004579	0.011
OPU 3	0.09008000	0.01800000	0.002
OPU 33	0.00816900	0.00259300	0.005
OPU 471	0.00044430	0.00008145	0.005
OPU 513	0.00038020	0.00007154	0.009
OPU 208	0.00037650	0.00007525	0.008
OPU 4	0.00031920	0.00005460	0.003
OPU 206	0.00025090	0.00005015	0.003
OPU 281	0.00274400	0.00046390	0.001
OPU 234	0.00241400	0.00041980	0.001
OPU 243	0.00172200	0.00029670	0.001
OPU 433	0.00140500	0.00024010	0.001
OPU 374	0.00134200	0.00022770	0.001
OPU 62	0.00101200	0.00018220	0.001
OPU 332	0.00069900	0.00011870	0.001
OPU 257	0.00057510	0.00009782	0.001
OPU 226	0.00057440	0.00009760	0.001
OPU 473	0.00050430	0.00009872	0.001
OPU 435	0.00044730	0.00007652	0.001
OPU 376	0.00044670	0.00007707	0.001
OPU 233	0.00038380	0.00006475	0.001
OPU 213	0.00031760	0.00005675	0.001
OPU 247	0.00025580	0.00004317	0.001
OPU 244	0.00025540	0.00004416	0.001

!

E:

TA393	Average	St. Dev	p-value
OPU 574	0.00058230	0.00012470	0.001
OPU 505	0.00302800	0.00060830	0.002
OPU 504	0.00110700	0.00022130	0.002
OPU 238	0.00081880	0.00015990	0.002
OPU 280	0.00044710	0.00008747	0.002
OPU 629	0.00036950	0.00007337	0.002
OPU 633	0.00032460	0.00010970	0.002
OPU 191	0.00036750	0.00007603	0.004
OPU 713	0.00083600	0.00032540	0.006
OPU 636	0.00044400	0.00014840	0.006
OPU 37	0.00148500	0.00043150	0.015
OPU 208	0.00029240	0.00006632	0.021
OPU 613	0.00156200	0.00077160	0.024
OPU 207	0.00057790	0.00013130	0.027
OPU 116	0.01276000	0.00729500	0.033
OPU 684	0.00047450	0.00023970	0.033
OPU 681	0.00045780	0.00018010	0.038
OPU 366	0.00027750	0.00008418	0.045

!

Column “Average” makes reference to the average contribution of every OPU to overall dissimilarity and column “St. Dev” to the standard deviation of this contribution. The p-value makes reference to the significance of this contribution.

Table S5: List of all 757 OPUs and their abundance (Data Sheet 2)

Table S6: List of 42 core OPUs and relative abundance (Data Sheet 3)

Figure Legends

Figure S1. Error rate and rarefaction curve for 16S rRNA sequences

Figure S2. Non-metric Multidimensional Scaling (NMDs) based on OPU abundances. All samples found inside the red circle constitute biological replicates at a confidence level of 99.95%. Samples that remain on the outside present a different distribution, as indicated by a Kruskal-wallis test (p-value < 0.05).

Figure S3. Microbiome profile at Phylum, Class, Order, Family and Genus categories of the Antelope. Phylum: of 24 phyla, only 8 with 0.5% total reads were displayed; Class: of 38 classes classified, only 8 with >0.5% total reads were displayed, 0.62% total reads were unknown class. Order: of 67 orders classified, only 7 with > 0.5% total reads were displayed, 5.99% total reads were unknown order. Family: of 122 families classified, only 5 with > 1% total reads were displayed, 10.60% total reads were unknown family. Genus: of 252 genera classified, only 3 with >1% total reads displayed, 73.06% total reads were unknown genus.

Figure S4. Phylogeny of core OPUs assigned as uncultured *Christensenellaceae*
(A) Phylogenetic comparison was carried using OPU 151, OPU 160, OPU 164, and only one genus and species named *Christensenella minuta* reported in *Christensenellaceae* family. The 16S rRNA sequences of *Christensenellaceae* from human were downloaded and included in the analysis [20] to determine phylogenetic relationship of the shared taxa in human and antelope. The type strains in other families within Clostridiales order were used as outgroup. Three OPUs are grouped in red box. Maximum likelihood phylogenies were constructed using RAxML (100 runs) as described in Methods. **(B)** An extension of phylogenetic branch clustered into OPU 160. Sequences from human

are indicated in red color.

Figure S5. Phylogeny of core OPU assigned as uncultured *Lachnospiraceae*

Phylogenetic comparison was carried using seven OPUs (OPU 612, OPU 617, OPU 618, OPU 664, OPU 666, OPU 668, and OPU 704) and representatives of type strains in *Lachnospiraceae* family. The 16S rRNA sequences of *Lachnospiraceae* that were shared with human and associated with health or longevity were downloaded and included in analysis. Type strains from other families within the Clostridiales order were used as outgroup. Maximum likelihood phylogenies were constructed using RAxML (100 runs). Seven OPU were group in red box. OTUs in a specific OPU group identified in this study were showed in red. Sequences from humans were colored in blue.

Figure S6. Phylogeny of core taxa assigned as *Ruminococcaceae* in antelope and human

Phylogenetic comparison was carried using representative sequences assigned as *Ruminococcaceae* in this study, type strains in *Ruminococcaceae* family, and sequences of *Ruminococcaceae* from humans. Maximum likelihood phylogenies were constructed using RAxML (100 runs). Sequences from humans were colored in blue.

Figure S7. Phylogeny of core taxa assigned as *Akkermansia* in antelope and human

Phylogenetic comparison was carried using representative sequences assigned as *Akkermansia* in this study, type strains in *Akkermansia* genus, and sequences of *Akkermansia* from humans. Two type strains from *Verrucomicrobiaceae* family were used as outgroup. Maximum likelihood phylogenies were constructed using RAxML (100 runs). Sequences from humans were colored in red.

Figure S1

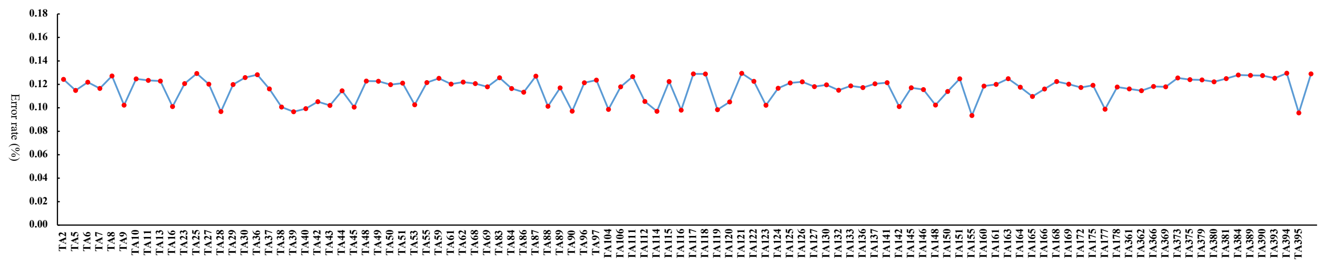

A

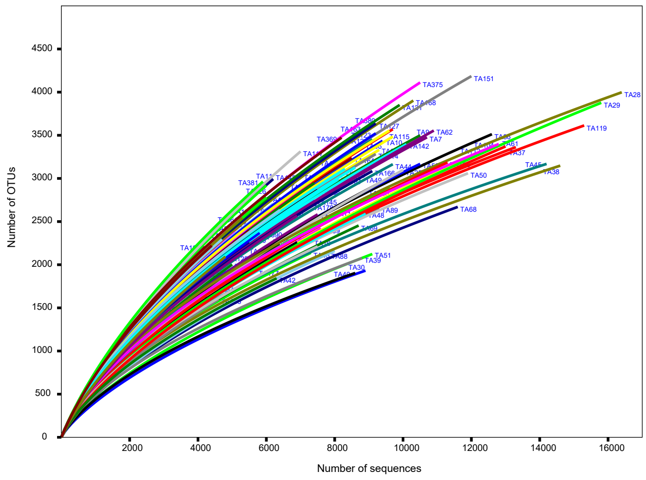

B

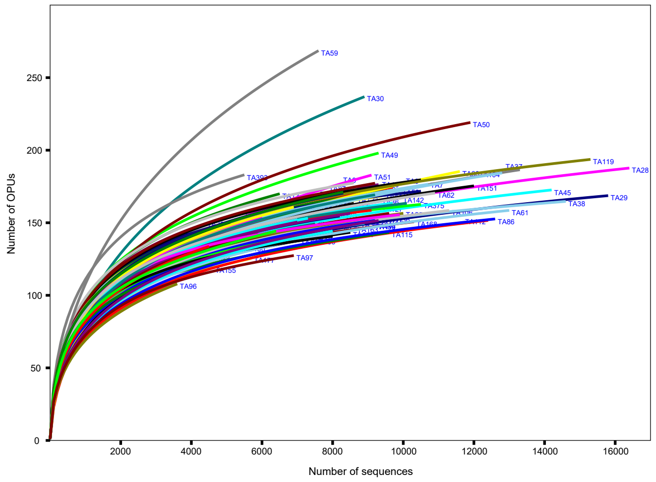

C

Figure S2

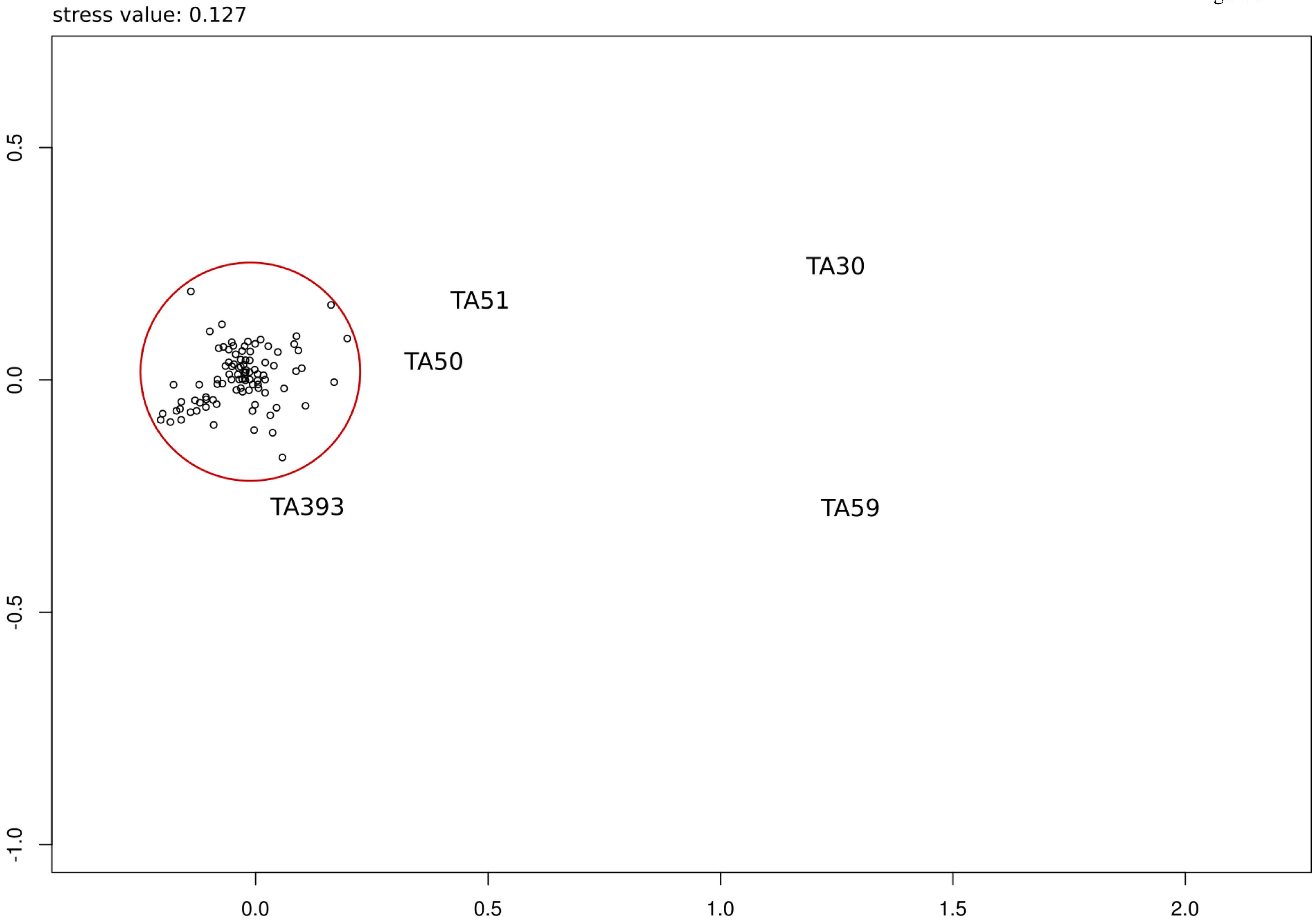
stress value: 0.127
TA30
TA51
TA50
TA393
TA59
0.5
0.0
-0.5
-1.0
0.0
0.5
1.0
1.5
2.0

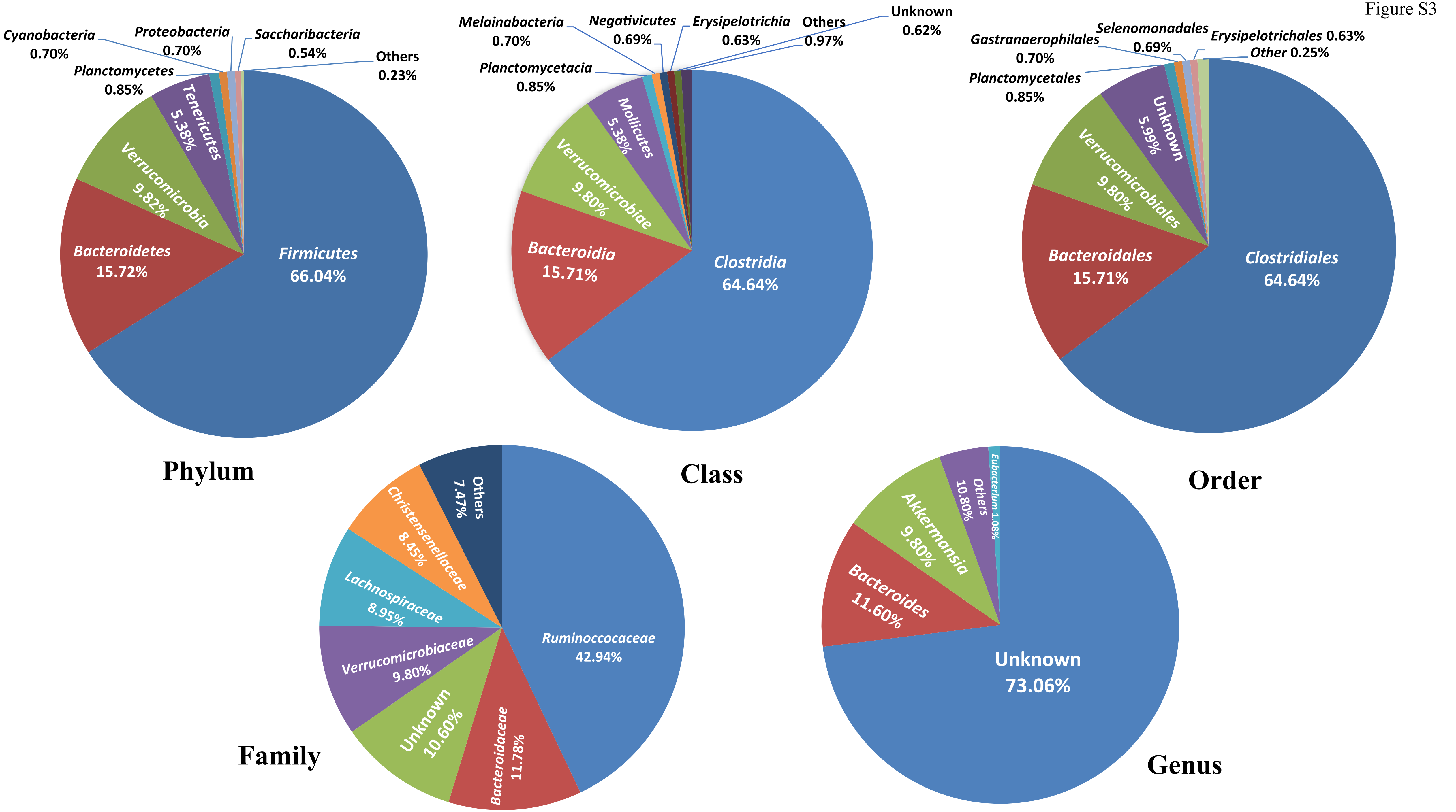
Cyanobacteria 0.70%
Proteobacteria 0.70%
Saccharibacteria 0.54%
Others 0.23%
Planctomycetes 0.85%
Tenericutes 5.38%
Verrucomicrobia 9.82%
Bacteroidetes 15.72%
Firmicutes 66.04%
Phylum
Melainabacteria 0.70%
Negativicutes 0.69%
Erysipelotrichia 0.63%
Others 0.97%
Unknown 0.62%
Planctomycetacia 0.85%
Mollicutes 5.38%
Verrucomicrobiae 9.80%
Bacteroidia 15.71%
Clostridia 64.64%
Class
Gastranaerophilales 0.70%
Selenomonadales 0.69%
Erysipelotrichales 0.63%
Other 0.25%
Planctomycetales 0.85%
Unknown 5.99%
Verrucomicrobiales 9.80%
Bacteroidales 15.71%
Clostridiales 64.64%
Order
Others 7.47%
Christensenellaceae 8.45%
Lachnospiraceae 8.95%
Verrucomicrobiaceae 9.80%
Unknown 10.60%
Bacteroidaceae 11.78%
Ruminoccocaceae 42.94%
Family
Eubacterium 1.08%
Others 10.80%
Akkermansia 9.80%
Bacteroides 11.60%
Unknown 73.06%
Genus

Figure S4

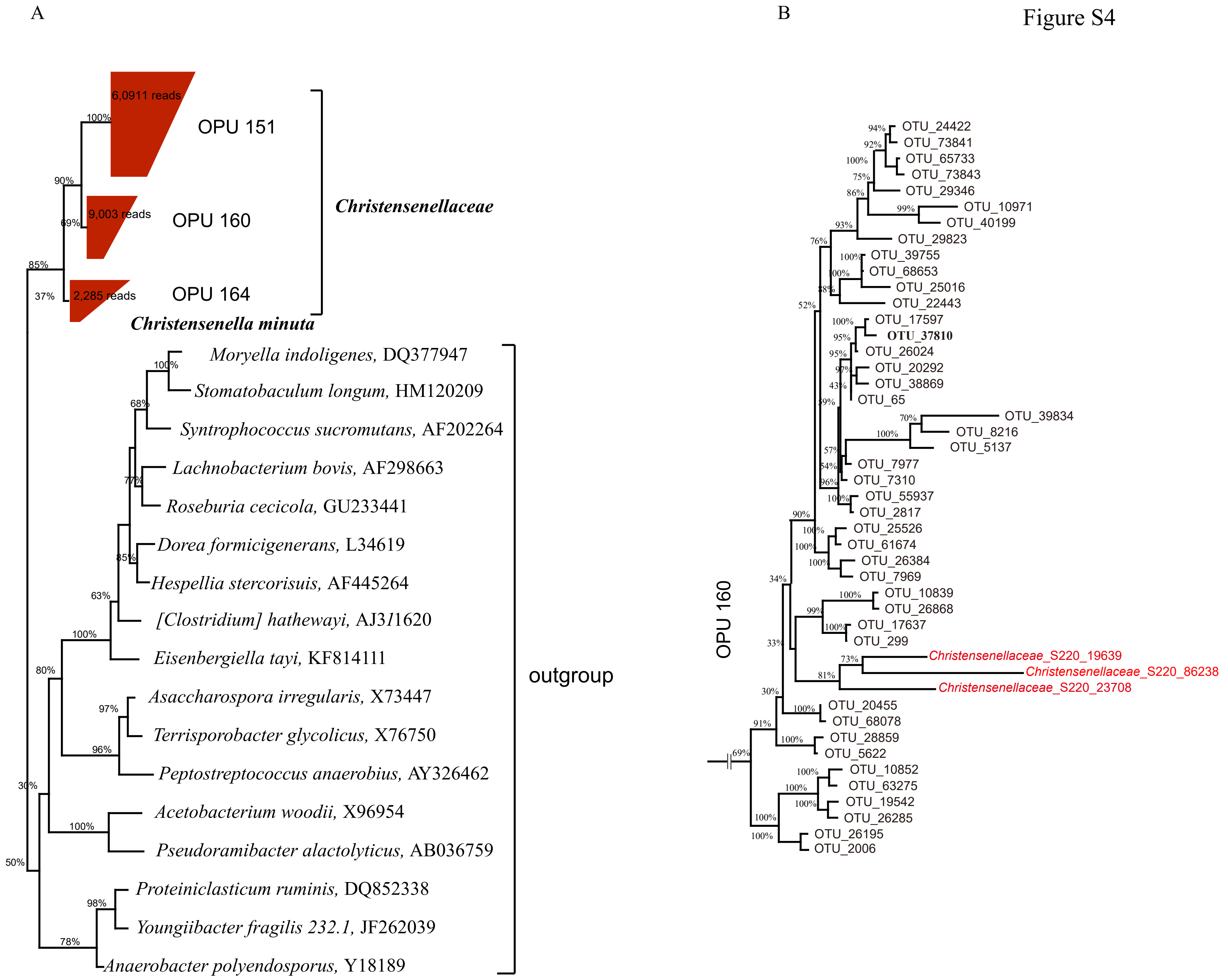

A
6,0911 reads
OPU 151
9,003 reads
OPU 160
2,285 reads
OPU 164
Christensenellaceae
Christensenella minuta
100%
90%
69%
85%
37%
Moryella indoligenes, DQ377947
Stomatobaculum longum, HM120209
Syntrophococcus sucromutans, AF202264
Lachnobacterium bovis, AF298663
Roseburia cecicola, GU233441
Dorea formicigenerans, L34619
Hespellia stercorisuis, AF445264
[Clostridium] hathewayi, AJ311620
Eisenbergiella tayi, KF814111
Asaccharospora irregularis, X73447
Terrisporobacter glycolicus, X76750
Peptostreptococcus anaerobius, AY326462
Acetobacterium woodii, X96954
Pseudoramibacter alactolyticus, AB036759
Proteiniclasticum ruminis, DQ852338
Youngiibacter fragilis 232.1, JF262039
Anaerobacter polyendosporus, Y18189
outgroup
100%
68%
77%
85%
63%
100%
80%
97%
96%
30%
100%
50%
98%
78%
B
OPU 160
OTU_24422
OTU_73841
OTU_65733
OTU_73843
OTU_29346
OTU_10971
OTU_40199
OTU_29823
OTU_39755
OTU_68653
OTU_25016
OTU_22443
OTU_17597
OTU_37810
OTU_26024
OTU_20292
OTU_38869
OTU_65
OTU_39834
OTU_8216
OTU_5137
OTU_7977
OTU_7310
OTU_55937
OTU_2817
OTU_25526
OTU_61674
OTU_26384
OTU_7969
OTU_10839
OTU_26868
OTU_17637
OTU_299
Christensenellaceae_S220_19639
Christensenellaceae_S220_86238
Christensenellaceae_S220_23708
OTU_20455
OTU_68078
OTU_28859
OTU_5622
OTU_10852
OTU_63275
OTU_19542
OTU_26285
OTU_26195
OTU_2006
94%
92%
100%
75%
86%
99%
93%
76%
100%
88%
52%
100%
95%
95%
43%
70%
100%
57%
54%
96%
90%
100%
100%
100%
34%
100%
99%
100%
33%
73%
81%
30%
100%
91%
100%
69%
100%
100%
100%
100%
100%

Figure S5

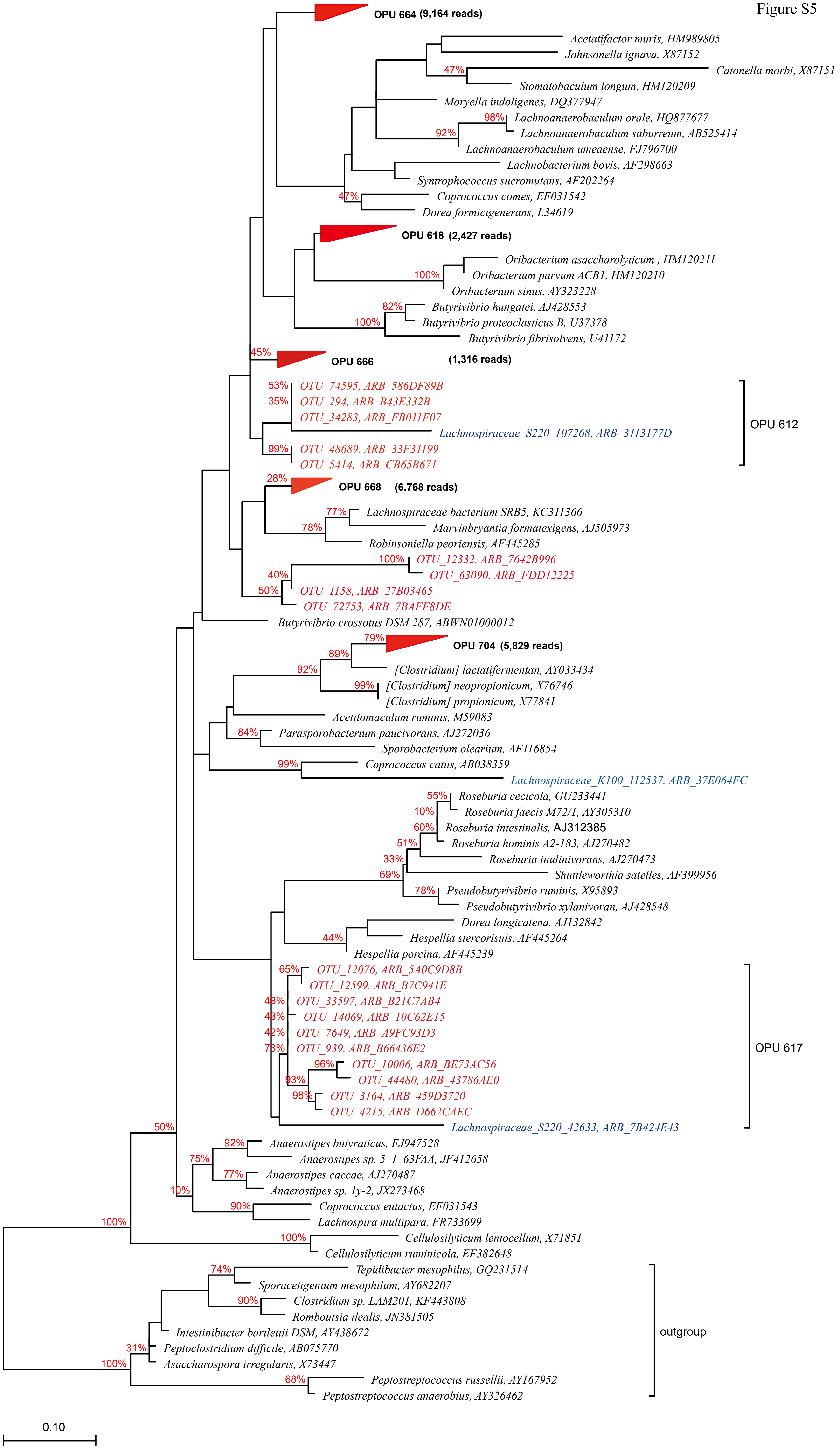
OPU 664 (9,164 reads)
Acetatifactor muris, HM989805
Johnsonella ignava, X87152
47%
Catonella morbi, X87151
Stomatobaculum longum, HM120209
Moryella indoligenes, DQ377947
98%
Lachnoanaerobaculum orale, HQ877677
92%
Lachnoanaerobaculum saburreum, AB525414
Lachnoanaerobaculum umeaense, FJ796700
Lachnobacterium bovis, AF298663
Syntrophococcus sucromutans, AF202264
47%
Coprococcus comes, EF031542
Dorea formicigenerans, L34619
OPU 618 (2,427 reads)
Oribacterium asaccharolyticum , HM120211
100%
Oribacterium parvum ACB1, HM120210
Oribacterium sinus, AY323228
82%
Butyrivibrio hungatei, AJ428553
100%
Butyrivibrio proteoclasticus B, U37378
Butyrivibrio fibrisolvens, U41172
45%
OPU 666 (1,316 reads)
53% OTU_74595, ARB_586DF89B
35% OTU_294, ARB_B43E332B
OTU_34283, ARB_FB011F07
Lachnospiraceae_S220_107268, ARB_3113177D
99% OTU_48689, ARB_33F31199
OTU_5414, ARB_CB65B671
OPU 612
28%
OPU 668 (6.768 reads)
77%
Lachnospiraceae bacterium SRB5, KC311366
78%
Marvinbryantia formatexigens, AJ505973
Robinsoniella peoriensis, AF445285
100% OTU_12332, ARB_7642B996
40%
OTU_63090, ARB_FDD12225
50% OTU_1158, ARB_27B03465
OTU_72753, ARB_7BAFF8DE
Butyrivibrio crossotus DSM 287, ABWN01000012
79%
OPU 704 (5,829 reads)
89%
[Clostridium] lactatifermentan, AY033434
92%
99% [Clostridium] neopropionicum, X76746
[Clostridium] propionicum, X77841
Acetitomaculum ruminis, M59083
84% Parasporobacterium paucivorans, AJ272036
Sporobacterium olearium, AF116854
99% Coprococcus catus, AB038359
Lachnospiraceae_K100_112537, ARB_37E064FC
55% Roseburia cecicola, GU233441
10% Roseburia faecis M72/1, AY305310
60% Roseburia intestinalis, AJ312385
51% Roseburia hominis A2-183, AJ270482
33% Roseburia inulinivorans, AJ270473
69% Shuttleworthia satelles, AF399956
78% Pseudobutyrivibrio ruminis, X95893
Pseudobutyrivibrio xylanivoran, AJ428548
Dorea longicatena, AJ132842
44% Hespellia stercorisuis, AF445264
Hespellia porcina, AF445239
65% OTU_12076, ARB_5A0C9D8B
OTU_12599, ARB_B7C941E
48% OTU_33597, ARB_B21C7AB4
43% OTU_14069, ARB_10C62E15
42% OTU_7649, ARB_A9FC93D3
73% OTU_939, ARB_B66436E2
96% OTU_10006, ARB_BE73AC56
93% OTU_44480, ARB_43786AE0
98% OTU_3164, ARB_459D3720
OTU_4215, ARB_D662CAEC
Lachnospiraceae_S220_42633, ARB_7B424E43
OPU 617
50%
92% Anaerostipes butyraticus, FJ947528
75% Anaerostipes sp. 5_1_63FAA, JF412658
77% Anaerostipes caccae, AJ270487
10% Anaerostipes sp. 1y-2, JX273468
90% Coprococcus eutactus, EF031543
Lachnospira multipara, FR733699
100%
100% Cellulosilyticum lentocellum, X71851
Cellulosilyticum ruminicola, EF382648
74% Tepidibacter mesophilus, GQ231514
Sporacetigenium mesophilum, AY682207
90% Clostridium sp. LAM201, KF443808
Romboutsia ilealis, JN381505
Intestinibacter bartlettii DSM, AY438672
31% Peptoclostridium difficile, AB075770
100% Asaccharospora irregularis, X73447
68% Peptostreptococcus russellii, AY167952
Peptostreptococcus anaerobius, AY326462
outgroup
0.10

Figure S6

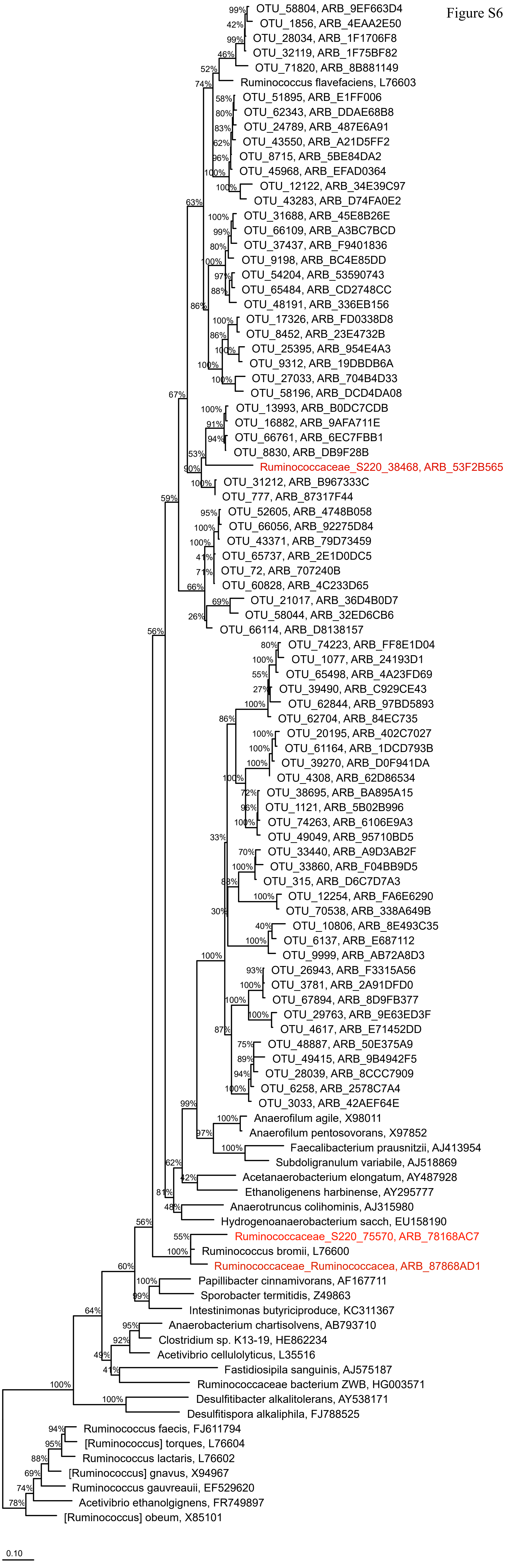
OTU_58804, ARB_9EF663D4
OTU_1856, ARB_4EAA2E50
OTU_28034, ARB_1F1706F8
OTU_32119, ARB_1F75BF82
OTU_71820, ARB_8B881149
Ruminococcus flavefaciens, L76603
OTU_51895, ARB_E1FF006
OTU_62343, ARB_DDAE68B8
OTU_24789, ARB_487E6A91
OTU_43550, ARB_A21D5FF2
OTU_8715, ARB_5BE84DA2
OTU_45968, ARB_EFAD0364
OTU_12122, ARB_34E39C97
OTU_43283, ARB_D74FA0E2
OTU_31688, ARB_45E8B26E
OTU_66109, ARB_A3BC7BCD
OTU_37437, ARB_F9401836
OTU_9198, ARB_BC4E85DD
OTU_54204, ARB_53590743
OTU_65484, ARB_CD2748CC
OTU_48191, ARB_336EB156
OTU_17326, ARB_FD0338D8
OTU_8452, ARB_23E4732B
OTU_25395, ARB_954E4A3
OTU_9312, ARB_19DBDB6A
OTU_27033, ARB_704B4D33
OTU_58196, ARB_DCD4DA08
OTU_13993, ARB_B0DC7CDB
OTU_16882, ARB_9AFA711E
OTU_66761, ARB_6EC7FBB1
OTU_8830, ARB_DB9F28B
Ruminococcaceae_S220_38468, ARB_53F2B565
OTU_31212, ARB_B967333C
OTU_777, ARB_87317F44
OTU_52605, ARB_4748B058
OTU_66056, ARB_92275D84
OTU_43371, ARB_79D73459
OTU_65737, ARB_2E1D0DC5
OTU_72, ARB_707240B
OTU_60828, ARB_4C233D65
OTU_21017, ARB_36D4B0D7
OTU_58044, ARB_32ED6CB6
OTU_66114, ARB_D8138157
OTU_74223, ARB_FF8E1D04
OTU_1077, ARB_24193D1
OTU_65498, ARB_4A23FD69
OTU_39490, ARB_C929CE43
OTU_62844, ARB_97BD5893
OTU_62704, ARB_84EC735
OTU_20195, ARB_402C7027
OTU_61164, ARB_1DCD793B
OTU_39270, ARB_D0F941DA
OTU_4308, ARB_62D86534
OTU_38695, ARB_BA895A15
OTU_1121, ARB_5B02B996
OTU_74263, ARB_6106E9A3
OTU_49049, ARB_95710BD5
OTU_33440, ARB_A9D3AB2F
OTU_33860, ARB_F04BB9D5
OTU_315, ARB_D6C7D7A3
OTU_12254, ARB_FA6E6290
OTU_70538, ARB_338A649B
OTU_10806, ARB_8E493C35
OTU_6137, ARB_E687112
OTU_9999, ARB_AB72A8D3
OTU_26943, ARB_F3315A56
OTU_3781, ARB_2A91DFD0
OTU_67894, ARB_8D9FB377
OTU_29763, ARB_9E63ED3F
OTU_4617, ARB_E71452DD
OTU_48887, ARB_50E375A9
OTU_49415, ARB_9B4942F5
OTU_28039, ARB_8CCC7909
OTU_6258, ARB_2578C7A4
OTU_3033, ARB_42AEF64E
Anaerofilum agile, X98011
Anaerofilum pentosovorans, X97852
Faecalibacterium prausnitzii, AJ413954
Subdoligranulum variabile, AJ518869
Acetanaerobacterium elongatum, AY487928
Ethanoligenens harbinense, AY295777
Anaerotruncus colihominis, AJ315980
Hydrogenoanaerobacterium sacch, EU158190
Ruminococcaceae_S220_75570, ARB_78168AC7
Ruminococcus bromii, L76600
Ruminococcaceae_Ruminococcacea, ARB_87868AD1
Papillibacter cinnamivorans, AF167711
Sporobacter termitidis, Z49863
Intestinimonas butyriciproduce, KC311367
Anaerobacterium chartisolvens, AB793710
Clostridium sp. K13-19, HE862234
Acetivibrio cellulolyticus, L35516
Fastidiosipila sanguinis, AJ575187
Ruminococcaceae bacterium ZWB, HG003571
Desulfitibacter alkalitolerans, AY538171
Desulfitispora alkaliphila, FJ788525
Ruminococcus faecis, FJ611794
[Ruminococcus] torques, L76604
Ruminococcus lactaris, L76602
[Ruminococcus] gnavus, X94967
Ruminococcus gauvreauii, EF529620
Acetivibrio ethanolgignens, FR749897
[Ruminococcus] obeum, X85101
0.10

Figure S7

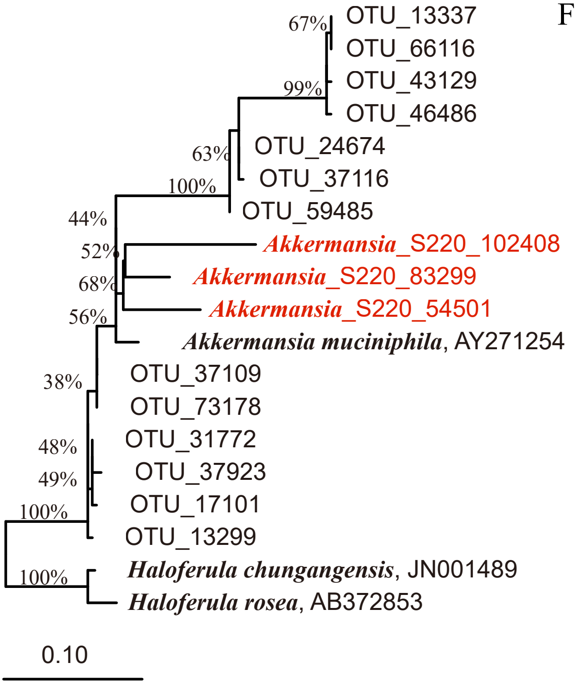

67%
OTU_13337
OTU_66116
99%
OTU_43129
OTU_46486
63%
OTU_24674
OTU_37116
100%
OTU_59485
44%
Akkermansia_S220_102408
52%
Akkermansia_S220_83299
68%
Akkermansia_S220_54501
56%
Akkermansia muciniphila, AY271254
38%
OTU_37109
OTU_73178
48%
OTU_31772
49%
OTU_37923
OTU_17101
100%
OTU_13299
100%
Haloferula chungangensis, JN001489
Haloferula rosea, AB372853
0.10
